# Self-Rated Health and Persistent Symptoms Among Adults Reporting Previous SARS-CoV-2 Infection: A Cross-Sectional Study

**DOI:** 10.3390/jcm15197342

**Published:** 2026-09-22

**Authors:** Dorota Weber, Robert Jan Łuczyk, Anna Pacian, Patrycja Brus, Kamil Sikora, Marta Łuczyk, Anna Charuta

**Affiliations:** 1 Faculty of Health Sciences, University of Economics and Innovation in Lublin, 20-209 Lublin, Poland; 2 Institute of Health Sciences, Faculty of Medical and Health Sciences, University of Siedlce, 08-110 Siedlce, Poland; robert.luczyk@uws.edu.pl (R.J.Ł.); anna.charuta@uws.edu.pl (A.C.); 3 Department of Health Education, Faculty of Health Sciences, Medical University of Lublin, 20-081 Lublin, Poland; anna.pacian@umlub.edu.pl (A.P.); patrycja.brus24@wp.pl (P.B.); 4 Department of Internal Medicine and Internal Nursing, Chair of Preventive Nursing, Faculty of Health Sciences, Medical University of Lublin, 20-093 Lublin, Poland; kamil.sikora@umlub.edu.pl; 5 Long-Term Care Nursing Department, Chair of Preventive Nursing, Faculty of Health Sciences, Medical University of Lublin, Ul. Chodźki 7, 20-093 Lublin, Poland; marta.luczyk@umlub.edu.pl

**Keywords:** SARS-CoV-2, COVID-19, persistent symptoms, self-rated health, psychological distress, general health questionnaire

## Abstract

**Background/Objectives**: Health self-assessment captures perceived physical and psychological well-being. This study described current and recalled pre-infection health ratings, persistent symptoms, and sociodemographic and clinical correlates among adults reporting previous severe acute respiratory syndrome coronavirus 2 (SARS-CoV-2) infection. **Methods**: A cross-sectional survey of 120 adults was conducted at University Clinical Hospital No. 4 in Lublin, Poland, and online between January and April 2026. An author-designed questionnaire assessed sociodemographic and clinical characteristics, current and recalled pre-infection general health, and symptoms following coronavirus disease 2019 (COVID-19). The 30-item General Health Questionnaire (GHQ-30) assessed current psychological symptoms. Both general health ratings were obtained in the same survey. Bivariate analyses used non-parametric tests and chi-square tests, with *p* < 0.05 as the nominal significance threshold. **Results**: Very good health was reported by 44.2% when recalling pre-infection health and by 28.3% for current health. Current health ratings were associated with age, education, employment, financial situation, and reported disease severity (all *p* < 0.001). The most commonly reported current complaints were fatigue/weakness (30.8%), cognitive disturbances (‘brain fog’; 17.5%), and dyspnoea (16.7%). Mean normalised GHQ-30 domain scores were 37.1/100 for anxiety and depression, 39.0/100 for general functioning, and 30.5/100 for interpersonal relationships. Poorer financial situation and more COVID-19 hospitalisations showed small positive correlations with poorer GHQ-30 scores (R = 0.19–0.27). Vaccine dose count showed a small correlation with general functioning (R = 0.18; *p* = 0.045). **Conclusions**: Current health was rated less favourably than recalled pre-infection health in this sample. Recall bias, selection effects, and unmeasured or uncontrolled confounding prevent attribution of this difference to infection. The exploratory associations do not establish independent risk factors, clinical diagnoses, or population prevalence.

## 1. Introduction

Health is defined by the World Health Organization as a state of complete physical, mental, and social well-being, and not merely the absence of disease or infirmity [1]. This holistic conception of health is shaped by a range of determinants, including lifestyle, environment, social and economic conditions, genetic predispositions, and access to healthcare [2].

Coronavirus disease 2019 (COVID-19), caused by severe acute respiratory syndrome coronavirus 2 (SARS-CoV-2), rapidly evolved from a localised outbreak into a global pandemic, affecting populations on all inhabited continents [3,4,5]. Beyond its acute respiratory manifestations, COVID-19 affects multiple organ systems, producing respiratory, cardiovascular, neurological, endocrinological, and psychosocial complications [6,7]. Symptoms may persist beyond the acute phase; however, definitions of long COVID and post-COVID-19 syndrome differ in their duration thresholds, symptom requirements, functional criteria, and exclusion of alternative explanations. These differences limit comparisons between studies [8].

Persistent symptoms reported after COVID-19 are heterogeneous and frequently encompass fatigue, dyspnoea, cognitive disturbances (commonly referred to as ‘brain fog’), disturbances of smell or taste, sleep disorders, and mood disturbances such as anxiety and depression [9,10,11,12,13]. Severity of the acute disease course, requirement for hospitalisation, and pre-existing comorbidities have been investigated in relation to persistent symptoms [14,15]. Psychological symptoms and socioeconomic circumstances are also associated with long COVID outcomes, although their temporal and causal relationships are not fully established [16,17].

Despite a growing body of international literature, data characterising perceived health after COVID-19 in Polish settings remain limited, and published findings vary substantially according to disease severity, timing of assessment, and study population characteristics. Descriptive studies can identify reported concerns and generate hypotheses for further research, provided that sample-specific findings are distinguished from population estimates and from effects attributable to infection.

The aim of this cross-sectional study was to describe health status among adults reporting previous SARS-CoV-2 infection, with particular attention to: (1) current and retrospectively recalled pre-infection self-rated general health, obtained at the same assessment; (2) the frequency and pattern of self-reported persistent symptoms; (3) current psychological symptoms; and (4) unadjusted associations with sociodemographic and clinical characteristics.

## 2. Materials and Methods

### 2.1. Study Design and Setting

A cross-sectional study was conducted between January and April 2026 among adults reporting previous SARS-CoV-2 infection. Hospital-based recruitment was conducted continuously among adults hospitalised in COVID-19 or internal-medicine wards or presenting to hospital-affiliated outpatient clinics; medical staff (physicians or nurses) invited eligible participants. The hospital questionnaire was administered on paper and was completed independently or with assistance. A separate online arm used voluntary snowball sampling and announcements in social-media groups, long-COVID support groups, and university or medical forums. The online questionnaire was completed anonymously using Google Forms. These dates describe questionnaire administration, rather than a common period of infection. Participation was voluntary and anonymous; participants were informed of the study objectives and of their right to withdraw at any stage without providing a reason. Current and recalled pre-infection health were assessed in the same questionnaire; no prospective pre-infection health measurement was available.

### 2.2. Participants

Eligibility criteria comprised: age ≥ 18 years, a reported history of SARS-CoV-2 infection, and provision of informed consent. For hospital-based participants, previous infection was confirmed from medical documentation showing a positive reverse-transcription polymerase chain reaction (RT-PCR) or rapid antigen test performed in hospital or laboratory settings during recruitment or a previous hospitalisation. For online participants, previous infection was accepted on the basis of the participant’s declaration; no laboratory or medical records were independently inspected for the online arm. These eligibility pathways concern the previous infection, rather than a clinical diagnosis of the cause of current symptoms. A total of 120 questionnaires were included in the final analysis. The mean age of participants was 31.5 years (standard deviation [SD] = 14.9; range 18–82 years); 67.5% were female, and 32.5% were male. The sample describes the respondents who participated and should not be regarded as representative of all Polish adults with previous COVID-19.

### 2.3. Data Collection Instruments

Data were collected using two instruments. The first was an author-designed, Polish-language questionnaire comprising 21 items in two parts: (1) sociodemographic characteristics (age, sex, education, employment status, marital status, place of residence, financial situation) and a clinical history of COVID-19 (number of infections, time since last infection, vaccination status, chronic diseases, disease severity, and history of COVID-19-related hospitalisation); and (2) retrospectively recalled pre-infection and current self-rated general health, and the presence, type, and severity of persistent health complaints. This component provided descriptive information; it was not a diagnostic instrument for post-COVID-19 condition. The administered form should be supplied as supplementary material so that its wording, response options, language and formatting can be evaluated. No evidence of reliability or validity of these study-specific items is reported.

The survey distinguished current complaints from symptoms recalled at any time since recovery from the acute illness. In this report, ‘persistent symptoms’ is a descriptive term for respondents’ complaints, rather than confirmation of a standardised post-COVID-19 diagnosis. Time since infection alone does not establish symptom duration, continuity, or exclusion of alternative causes.

The second instrument was the 30-item General Health Questionnaire (GHQ-30), developed by David Goldberg and administered in a Polish-language adaptation, to assess current psychological symptoms. The Polish factor-structure evidence used to support this instrument is reported by Frydecka et al. [18]. The instrument comprises 30 items rated on a four-point Likert scale using the 0–1–2–3 scoring convention, addressing the respondent’s experience over recent weeks relative to their usual state. Results were reported for three domains: anxiety and depression, interpersonal relationships, and general functioning. Raw domain scores were normalised to a 0–100 scale, with higher scores indicating greater reported difficulties. The available materials do not establish the item key, normalisation formula or missing-item rule used for the reported domain scores; therefore, these scores are interpreted descriptively and do not establish psychiatric diagnoses or validated mild, moderate, or severe categories.

Relevant Polish psychometric evidence comes from Frydecka et al., who studied 623 patients with neurotic disorders or somatic illnesses. They reported high internal consistency (Cronbach’s alpha = 0.97 in the Results) and a three-factor solution accounting for approximately 58% of variance, with domain labels corresponding to those used here [18]. This supports the relevance of the constructs, but does not validate the exact item allocation, normalisation, or diagnostic interpretation used in this post-infection sample. No study-specific reliability or criterion-validity estimates are reported.

### 2.4. Statistical Analysis

Categorical variables were described using frequencies and percentages; continuous variables were described using means, standard deviations (SD), medians, and minimum–maximum ranges. The normality of continuous variable distributions was assessed using the Shapiro–Wilk test; as the assumption of normality was not met throughout, non-parametric tests were applied. Between-group comparisons were performed using the Mann–Whitney U test (two groups) or the Kruskal–Wallis test (three or more groups). Associations between continuous and ordinal variables were assessed using the Spearman rank correlation coefficient (R). The chi-square test was applied to evaluate associations between categorical variables. The reported analyses were bivariate and did not adjust for potential confounders. The threshold *p* < 0.05 is treated as nominal, and findings are exploratory in view of multiple comparisons. The magnitude and direction of R are considered alongside *p* values; statistical significance alone does not establish clinical importance. No new statistical analyses were performed for this revision. Analyses were performed using StatSoft Statistica 13.1 PL (StatSoft, Inc., Tulsa, OK, USA) and Microsoft Excel.

The reported comparison categories were female/male; rural/urban residence; vocational/secondary/university education; single/married/cohabiting relationship status; employed/retired or pension recipient and unemployed/student or pupil; and very good/good/poor self-rated financial situation. The descriptive age groups were ≤20, 21–29, 30–39, 40–49, 50–59, and ≥60 years. Disease-severity categories were asymptomatic, mild, moderate, and severe. Current health categories were very good, good, average, poor, and very poor. Financial situation represents a subjective category, not a reported income threshold. Hospitalisations and vaccine doses were examined as counts in the reported Spearman correlations. Group-specific percentages and an overall test do not establish significant differences between particular pairs of groups.

### 2.5. Ethical Considerations

The study was conducted in accordance with the Declaration of Helsinki. As the survey was fully anonymous, non-interventional, and did not interfere with participants’ treatment or care, formal bioethics committee approval was not required under applicable institutional regulations; institutional permission was obtained from the hospital directorate. All participants provided informed consent prior to enrolment. The survey was fully anonymous.

## 3. Results

### 3.1. Sociodemographic and Clinical Characteristics

The study group comprised 120 individuals reporting previous COVID-19. The largest age group was 21–29 years (61.7%); women predominated (67.5%); the majority of participants were urban residents (65.8%) and held a university-level degree (57.5%). Almost half of respondents were single (49.2%), and approximately half were employed (45.8%), with a similar proportion of students/pupils (44.2%). The financial situation was rated as good by 70.0% of participants and as very good by 22.5%. Full sociodemographic characteristics are presented in Table 1. The predominance of young adults is a characteristic of this respondent sample and does not describe the age distribution of infections in the source population.

The majority of participants (70.8%) reported having contracted COVID-19 once; 22.5% twice; and 6.7% three times or more. The most frequently reported time elapsed since the last infection was more than 4 years (29.2%) or more than 5 years (23.3%). Regarding vaccination status, 38.3% of participants had received two doses, 31.7% were unvaccinated, 15.8% had received three doses, and 14.2% had received one dose. Chronic diseases were reported by 40.0% of participants, most frequently arterial hypertension (16.7%) and obesity (10.0%).

### 3.2. Current and Recalled Pre-Infection Self-Rated Health

When recalling their health before COVID-19, 44.2% of respondents selected ‘very good’, compared with 28.3% for current health. These are two ratings obtained at one assessment, rather than measurements collected before and after infection. The most common current health rating was ‘good’ (43.3%), followed by ‘average’ (23.3%) and ‘poor’ (5.0%); no respondent rated their current health as very poor. Current health ratings were associated with age (*p* < 0.0001), education (*p* < 0.001), employment (*p* < 0.001), financial situation (*p* < 0.0001), and reported disease severity (*p* < 0.0001) in the reported bivariate analyses. Descriptively, very good current health was more frequent among younger participants and those with a university-level education. The proportions reporting very good current health were 37.7% among students/pupils, 23.6% among employed participants, and 8.3% among those not working; 63.0%, 19.1%, and 11.1% among those reporting very good, good, and poor financial situations, respectively; and 50.0%, 32.5%, 17.3%, and 0% among those reporting asymptomatic, mild, moderate, and severe disease, respectively. These category-specific percentages do not establish significant pairwise differences. Sex, place of residence, and marital status were not significantly associated with current self-rated health (all *p* > 0.05).

### 3.3. Current Psychological Symptoms (GHQ-30)

Mean normalised GHQ-30 domain scores were 37.1 (SD = 17.1) for anxiety and depression, 30.5 (SD = 20.9) for interpersonal relationships, and 39.0 (SD = 18.4) for general functioning (Table 2). These descriptive scores do not provide estimates of the prevalence or clinical severity of anxiety or depressive disorders. Normalising domains to a common range does not establish that differences between their means are clinically comparable.

Age, sex, place of residence, education level, and employment status were not significantly associated with GHQ-30 scores in any domain (all *p* > 0.05). Selected associations with reported *p* < 0.05 are summarised in Table 3. Mean interpersonal relationship scores were 40.5 among cohabiting participants, 30.1 among married participants, and 26.0 among single participants. The reported overall marital-status comparison appears in Table 3; these descriptive means do not establish pairwise differences. Poorer financial situation showed small positive correlations with all three GHQ-30 domains (R = 0.26–0.27; all *p* ≤ 0.005). The number of COVID-19-related hospitalisations also showed small positive correlations with all three domains (R = 0.19–0.21; all *p* < 0.05). A higher number of vaccine doses received showed a small positive correlation with general functioning scores (R = 0.18; *p* = 0.045), whereas no statistically significant association was observed for the remaining two domains. These associations do not establish independent effects or clinically important differences.

### 3.4. Self-Reported Persistent Symptoms

Approximately half of respondents (50.8%) reported no current health complaints that they attributed to prior COVID-19. In the full sample, the most frequently reported current complaints were fatigue and weakness (30.8%), cognitive disturbances designated as ‘brain fog’ (17.5%), and dyspnoea (16.7%). When symptoms recalled at any time since recovery from the acute illness were considered, the most frequent were chronic fatigue (42.5%) and taste/smell disturbances (37.5%), followed by various types of pain (25.8%) and neurological symptoms (17.5%). Fewer than one-fifth of respondents (18.3%) reported the concurrent occurrence of more than three persistent symptoms. The reported bivariate findings also suggested an association of greater acute disease severity with persisting cough, weakness, fatigue, low mood, and anxiety. These self-reports do not establish that the symptoms were caused by SARS-CoV-2 or that respondents met a uniform clinical definition of post-COVID-19 condition.

## 4. Discussion

This study described current health and recalled pre-infection health among adults reporting previous SARS-CoV-2 infection, using an author-designed questionnaire and the GHQ-30. Very good health was reported by 44.2% when recalling pre-infection health and by 28.3% for current health. This difference is a comparison of contemporaneously collected current and retrospective ratings, not evidence of prospectively measured deterioration. The absence of an uninfected comparison group and a pre-infection baseline prevents attribution of the difference to COVID-19. In particular, ordinary ageing, new or pre-existing illness, and changing social circumstances may contribute over the several years since infection reported by many respondents.

Retrospective health assessment is vulnerable to inaccurate reconstruction of earlier health and to changes in how respondents interpret the rating scale [19]. Empirical work by Lawson et al. found closer agreement between prospective and recalled health at a group level than at an individual level in an orthopaedic setting with a short recall interval [20]. That result cannot establish the accuracy of multi-year recall in this sample. Current symptoms and psychological state may influence recollection; a lower current rating therefore indicates a reported contrast with remembered health, whose causes and accuracy remain uncertain.

The GHQ-30 results describe current psychological symptoms, but they cannot quantify the prevalence of clinically significant depression or anxiety without an appropriate validated threshold and diagnostic assessment. Renaud-Charest et al. reported heterogeneous estimates of depressive symptoms and clinical depression after COVID-19 across studies using different assessments [21]. Those prevalence estimates are not directly comparable with the present normalised domain means. Our results therefore describe reported difficulties rather than a mild-to-moderate level of psychiatric impairment.

For current complaints, fatigue/weakness, cognitive disturbances, and dyspnoea were most frequently reported; the higher frequencies of chronic fatigue and taste/smell disturbances refer to symptoms recalled at any time since recovery. Comparisons with other studies require attention to recruitment, infection ascertainment, symptom definitions, and assessment timing. Carfì et al. assessed previously hospitalised patients in an Italian post-acute service at a mean of approximately 60 days after symptom onset [22]. Davis et al. used an online survey distributed through support groups and social media, including people with suspected or confirmed infection and prolonged illness [10]. Chopra et al. assessed outcomes 60 days after hospital discharge [11]. These selected populations and assessment windows differ from our mixed hospital and online sample, in which many infections occurred several years earlier. Similar symptom patterns provide clinical context, but differences in reported frequencies cannot be interpreted as differences between national populations.

The heterogeneity described by Gutzeit et al. concerns both the definition of post-COVID syndromes and the symptoms and functional problems used to identify them [8]. Our questionnaire did not establish uniform clinical case ascertainment; its descriptive symptom frequencies should therefore not be presented as the prevalence of a standardised syndrome.

The recommendations of the European Network to improve diagnostic, treatment and healthcare for patients with persistent somatic symptoms (EURONET-SOMA) provide a framework for comparing assessment domains [23]. The domains described in our survey overlap partly with somatic symptoms, including fatigue, subjective cognitive complaints, psychological symptoms, general functioning, comorbidity history, and socioeconomic circumstances. However, a general health rating is not equivalent to a multidimensional quality-of-life measure, and relationship status or GHQ-30 interpersonal scores do not measure perceived social support directly. Dedicated assessments of post-exertional malaise, illness beliefs, behavioural responses, social support, and objective physical or cognitive status are not documented. This is a retrospective comparison of coverage, not evidence that the survey followed or fully implemented EURONET-SOMA.

A notable finding was the association between poorer self-rated financial situation and poorer current general health and GHQ-30 scores. The GHQ-30 correlations were small (R = 0.26–0.27), despite their nominal statistical significance. Tamim El Jarkass et al. reviewed 71 observational studies and reported associations of financial insecurity and lower education with adverse long COVID outcomes [17]. This provides context for our findings, but variation in case definitions and socioeconomic measures limits direct comparison. Financial situation may shape healthcare access and recovery conditions, while ill health may also reduce income or employment. Our bivariate cross-sectional analyses cannot distinguish these directions or determine whether financial situation is independently associated with health.

Engelmann et al. found that depression and anxiety were associated with long COVID, with evidence from both cross-sectional and prospective studies [16]. Their synthesis supports considering psychological symptoms alongside physical complaints, but our one-time GHQ-30 assessment cannot determine whether psychological difficulties preceded infection, followed persistent symptoms, or share other determinants. These associations do not demonstrate a psychological cause of the symptoms.

Alternative explanations also arise from general-population research. In a Czech adult survey, mental health contributed to self-rated general health after accounting for physical-health indicators and socioeconomic characteristics [24]. UK longitudinal research using the 12-item General Health Questionnaire documented socioeconomic inequalities in psychological distress before and during the pandemic [25]. The latter used a different GHQ version and was not a long COVID case sample. Thus, the associations observed here may reflect broader relationships between health, distress, and social circumstances that are not specific to previous infection.

Younger age was associated with more favourable current general health ratings, but this does not establish an age-specific effect of COVID-19. Likewise, the small correlations between hospitalisation count and GHQ-30 scores may reflect acute illness, pre-existing morbidity, or other shared factors. The small association between vaccine dose count and general functioning (R = 0.18; *p* = 0.045) is also unadjusted. Age, chronic illness, healthcare use, and the timing of vaccination and infection are possible alternative explanations; none were tested in an adjusted model. The association should not be interpreted as evidence that vaccination caused poorer functioning, nor as proof of any particular confounding mechanism.

### Limitations

Several limitations should be considered when interpreting these findings. First, both current and recalled pre-infection health were measured once. Recall bias and changes in the standards used to judge health may affect retrospective ratings, especially after a long interval [19,20]. The direction and magnitude of these biases cannot be determined here. Detailed pre-infection symptoms and prospectively measured mental health were unavailable, and there was no uninfected comparison group. Consequently, neither the difference between health ratings nor current symptoms can be attributed specifically to SARS-CoV-2.

Second, previous infection eligibility and clinical attribution of present symptoms are separate issues. A history of laboratory-confirmed or clinically diagnosed infection does not itself show that current fatigue, dyspnoea, or cognitive complaints are post-COVID sequelae. Formal clinical attribution of current complaints was absent for most participants. The lack of uniform symptom-duration criteria and systematic exclusion of alternative causes limits diagnostic specificity and comparison with studies using defined post-COVID cases.

Third, voluntary participation through hospital and online routes creates selection concerns. Online access, digital literacy, education, age, and interest in COVID-19 may influence participation. People with persistent complaints may be more motivated to respond, whereas severe illness or disability may impede participation; the net direction of bias is uncertain. Hospital recruitment may also select people with different morbidity and healthcare use. The predominance of respondents aged 21–29 years (61.7%) describes the sample, not all infections during the survey period. Without the number and characteristics of eligible nonrespondents and recruitment-channel counts, response rates and the extent of selection bias cannot be established. Findings should not be generalised to the Polish population.

Fourth, the sample size of 120 and small subgroups limit precision, while bivariate analyses leave confounding uncontrolled. Collected variables such as age, sex, education, employment, financial situation, chronic diseases, reported severity, time since infection, repeated infections, hospitalisation count, and vaccination history were not jointly modelled. Other potentially relevant factors are not characterised in sufficient detail here, including pre-infection psychological health, baseline function, treatment history, changes in income or work, and social support. Current psychological distress can also influence general health ratings independently of physical illness [24]. Independent associations, mediation, and temporal direction cannot be established.

Finally, multiple comparisons and selective presentation of associations with *p* < 0.05 increase the risk of overemphasising chance findings. Multiplicity-adjusted significance is not established by the reported presentation. Chi-square statistics, degrees of freedom, and standardised effect sizes are not reported, so categorical association magnitude cannot be assessed from *p* values alone; confidence intervals for the reported correlations are also unavailable. Non-significant results do not establish equivalence. The author-designed items lack reported validation evidence, and the transferability of the GHQ-30 domain scoring requires confirmation. Accordingly, the results are descriptive and hypothesis-generating.

## 5. Conclusions

1. Current general health was rated less favourably than recalled pre-infection health in this respondent sample. The two ratings were obtained in the same survey and do not demonstrate a longitudinal or causal effect of SARS-CoV-2 infection.
2. GHQ-30 domain scores describe current psychological difficulties; they do not establish clinical diagnoses or severity categories. Poorer financial situation and more COVID-19-related hospitalisations showed small, unadjusted correlations with higher scores.
3. The most frequently recalled symptoms at any time since recovery from the acute illness were chronic fatigue (42.5%) and taste/smell disturbances (37.5%), followed by various types of pain (25.8%) and neurological symptoms (17.5%).
4. Approximately half of participants (50.8%) reported no current symptoms attributed to prior COVID-19. In the full sample, the most common current complaints were fatigue and weakness (30.8%), cognitive disturbances (17.5%), and dyspnoea (16.7%). These frequencies are distinct from the retrospective symptom frequencies above.
5. Current self-rated health was associated with reported acute disease severity in bivariate analyses. This does not establish an independent effect after accounting for age, pre-existing health, or socioeconomic circumstances.
6. The findings identify reported health and psychosocial concerns within this selected sample and generate hypotheses for prospective studies with standardised assessment and adjustment for confounders. They do not establish population prevalence or the effectiveness of a screening or care pathway.

## Figures and Tables

**Table 1:** Sociodemographic characteristics of the study group (*n* = 120).

Variable	Category	*n*	%
Age (years)	≤20	9	7.5
	21–29	74	61.7
	30–39	11	9.2
	40–49	8	6.7
	50–59	9	7.5
	≥60	9	7.5
Sex	Female	81	67.5
	Male	39	32.5
Place of residence	Rural	41	34.2
	Urban	79	65.8
Education level	Vocational	9	7.5
	Secondary	42	35.0
	University/Higher	69	57.5
Marital status	Single	59	49.2
	Married	33	27.5
	Cohabiting (informal relationship)	28	23.3
Employment status	Employed	55	45.8
	Retired/pension and unemployed	9	10.0
	Student/pupil	53	44.2
Financial situation	Very good	27	22.5
	Good	84	70.0
	Poor	9	7.5

*n*—number of observations; %—percentage.

**Table 2:** General Health Questionnaire (GHQ-30) domain scores in the study group (*n* = 120; normalised 0–100 scale).

GHQ-30 Domain (0–100 Scale)	*n*	Mean	SD	Median	Range (Min–Max)
Anxiety and depression	120	37.1	17.1	36.7	3.3–93.3
Interpersonal relationships	120	30.5	20.9	25.0	0.0–100.0
General functioning	120	39.0	18.4	38.1	0.0–100.0

Higher scores indicate greater reported difficulties. GHQ-30: 30-item General Health Questionnaire; *n*: number of participants; SD: standard deviation; Min–Max: minimum–maximum. The normalised scores are descriptive; no clinical severity thresholds are applied.

**Table 3:** Selected bivariate associations with General Health Questionnaire (GHQ-30) domain scores and reported *p* < 0.05 (*n* = 120).

Variable	GHQ-30 Domain	Test Statistic	*p*-Value
Financial situation (poorer)	Anxiety and depression	Spearman R = 0.26	0.003
Financial situation (poorer)	Interpersonal relationships	Spearman R = 0.27	0.003
Financial situation (poorer)	General functioning	Spearman R = 0.26	0.005
Marital status (cohabiting)	Interpersonal relationships	Kruskal–Wallis H = 4.89	0.009
Number of COVID-19 hospitalisations	Anxiety and depression	Spearman R = 0.21	0.024
Number of COVID-19 hospitalisations	Interpersonal relationships	Spearman R = 0.19	0.037
Number of COVID-19 hospitalisations	General functioning	Spearman R = 0.21	0.021
Number of vaccine doses received	General functioning	Spearman R = 0.18	0.045

Only associations with reported *p* < 0.05 are shown. GHQ-30: 30-item General Health Questionnaire; *n*: number of participants; R: Spearman rank correlation coefficient; H: Kruskal–Wallis test statistic. The reported positive correlations (R = 0.18–0.27) are small in magnitude. Significance after correction for multiple testing is not claimed. The Kruskal–Wallis test concerns an overall group comparison and does not establish which pairs differ.

## Data Availability

The data presented in this study are available on request from the corresponding author.
